# Modulation of NLRP3 Inflammasome Attenuated Inflammatory Response Associated to Diarrhea-Predominant Irritable Bowel Syndrome

**DOI:** 10.3390/biomedicines8110519

**Published:** 2020-11-20

**Authors:** Sarah Adriana Scuderi, Giovanna Casili, Marika Lanza, Alessia Filippone, Irene Paterniti, Emanuela Esposito, Michela Campolo

**Affiliations:** Department of Chemical, Biological, Pharmaceutical and Environmental Sciences, University of Messina, Viale Ferdinando Stagno D’ Alcontres, 31-98166 Messina, Italy; sarascud@outlook.it (S.A.S.); gcasili@unime.it (G.C.); mlanza@unime.it (M.L.); afilippone@unime.it (A.F.); ipaterniti@unime.it (I.P.); michela.campolo@unime.it (M.C.)

**Keywords:** IBS-D, inflammasome, NLRP3, NF-κB, stress, intestinal permeability

## Abstract

Diarrhea-predominant irritable bowel syndrome (IBS-D) is a multifactorial chronic gastrointestinal disorder characterized by inflammation and immune response. In this context, NLRP3 over-activation is associated with a breakdown of enteric-immune balance related to IBS-D. The aim of this study was to evaluate the effect of the inflammasome inhibitor, BAY 11-7082, in a rat model of IBS-D. Syndrome was induced by intracolonic instillation of 1 mL 4% acetic acid at 8 cm proximal to the anus for 30 s and sacrificed 2 weeks after IBS-D induction. BAY 11-7082 (10 and 30 mg/kg) was administered daily by oral gavage. The results obtained showed that the treatment with BAY 11-7082 (30 mg/kg) significantly reduced tissue injury characterized by edema, neutrophil infiltration, and loss of colon structure. We demonstrated that BAY 11-7082 treatment inhibited NLRP3 inflammasome activation and NF-kB translocation, reducing inflammatory mediators. Moreover, treatment with BAY 11-7082 restored tight junction alteration following IBS-D induction and reduced the restraint stress. Taken together, our data demonstrate that IBS-D induced NLRP3 inflammasome pathway activation, accompanied by the production of proinflammatory response. The modulation of the inflammosome pathway with BAY 11-7082 inhibitor significantly reduced pathological signs of IBS-D, therefore, can be considered a valuable strategy to reduce the development of IBS-D.

## 1. Introduction

Irritable bowel syndrome (IBS) is a chronic gastrointestinal disorder characterized by recurrent abdominal pain [1]. Diarrhea-predominant irritable bowel syndrome (IBS-D) is generally reported as the most common subtype (28–46%) of IBS associated with abdominal bloating and watery stools [1,2]. The pathophysiology of IBS is unclear; however, several mechanisms have been proposed, including altered gastrointestinal (GI) microbiota, infectious gastroenteritis, visceral hypersensitivity, dysregulation of gut–brain axis, chronic stress, and genetic factors [3,4]. Several pharmacologic and non-pharmacologic treatments such as dietary modification and probiotics are available for IBS-D management, however there is still the need to identify new molecular targets and alternative treatments [5]. Recent studies revealed that inflammation plays a key role in IBS-D development, associated to intestinal dysfunction and increased intestinal epithelial cell (IEC) permeability [6,7]. The nucleotide-binding domain leucine-rich repeat-containing receptors (NLRs) are a family of intracellular innate immune receptors which play essential roles in the areas of intestinal mucosal immunity and inflammation [8]. Excessive NLR activation induces the assembly of multiprotein complexes known as inflammasomes which are involved in the inflammatory response [9]. Among the inflammasomes, one of the most studied is the NOD-like receptor family pyrin domain containing 3 (NLRP3) inflammasome [10]. NLRP3 plays a key role in the maintenance of intestinal homeostasis and modulates innate immune responses against commensal bacteria [11,12]. In presence of inflammatory stimuli, NLRP3 through the adapter protein apoptosis-associated speck-like protein (ASC) activates caspase-1 that in turn promotes the release of pro-inflammatory cytokines as interleukin-1β (IL-1β) and interleukin-18 (IL-18) involved in the inflammatory response [11]. Scientific evidence has demonstrated that NLRP3 over-activation during bowel inflammation could be associated with a breakdown of enteric immune balance, suggesting the involvement of NLRP3 in the pathogenesis of various immune-mediated diseases as IBS [11,13]. Therefore, the identification of NLRP3 inflammasome inhibitors results in important to design effective therapies against immune-mediated diseases [14]. In vivo and in vitro studies demonstrated that BAY 11-7082, a sulfonic derivative, is a selective inhibitor for NLRP3 inflammasome thanks its abilities to suppress the ATPase activity of NLRP3, required for its activation [14,15,16]. BAY 11-7082 exhibited several pharmacological activities including anticancer, neuroprotective, and anti-inflammatory effects as demonstrated in many studies [17,18,19,20]. Therefore, this study aimed to evaluate the effect of BAY 11-7082 in an in vivo model of IBS-D to study the involvement of inflammasome in the pathology and to identify a new possible treatment. 

## 2. Materials and Methods

### 2.1. Animals

Sprague–Dawley rats (Harlan, Milan, Italy) were housed in a controlled environment (22 ± 2 °C, 55 ± 15% relative humidity, 12 h light/dark cycle). After a one-week acclimation, rats were fed a standard diet and water. Animal experiments were in compliance with Italian regulations on protection of animals used for experimental and other scientific purposes (DM 116192) as well as EU regulations (OJ of EC L 358/1 18 December 1986). The animals used for this study were randomly selected from those suitable, available at that time.

### 2.2. Materials

All compounds were obtained from Sigma-Aldrich Company Ltd. (Milan, Italy). All stock solutions were prepared in non-pyrogenic saline (0.9% NaCl; Baxter, Liverpool, UK).

### 2.3. IBS-D Model 

After an overnight fast, the rats were anesthetized with ether, and IBS-D was induced by intracolonic, instillation of 1 mL 4% acetic acid at 8 cm proximal to the anus for 30 s. Then, 1 mL phosphate-buffered saline was instilled to dilute the acetic acid and flush the colon. The control animals were handled identically, except that 1 mL saline was instilled instead of 4% acetic acid. Rats were left to recover for 6 days. On the 7th day, the animals were assigned randomly to groups to receive the treatment BAY 11-7082 (Sigma-Aldrich^®^, catalog No.19542-67-7) at the doses of 10 and 30 mg/kg for 2 weeks daily by oral gavage. The animals were sacrificed after 2 weeks from the beginning of treatment with BAY 11-7082. Then, the colon tissues were taken for analysis.

### 2.4. Experimental Groups

The rats were randomly divided into 6 groups, as described below:

Group 1. Sham + vehicle (saline). Rats received saline for 2 weeks (N= 10).
Group 2. Sham + BAY 11-7082 10 mg/kg. Rats received BAY 11-7082 10 mg/kg for 2 weeks (N = 10).
Group 3. Sham + BAY 11-7082 30 mg/kg. Rats received BAY 11-7082 30 mg/kg for 2 weeks (N = 10).
Group 4. IBS-D + vehicle (saline). Rats received saline for 2 weeks after IBS-D induction (N = 10).
Group 5. IBS-D + BAY 11-7082 10 mg/kg. Rats received BAY 11-7082 10 mg/kg for 2 weeks after IBS-D induction (N = 10).
Group 6. IBS-D + BAY 11-7082 30 mg/kg. Rats received BAY 11-7082 30 mg/kg for 2 weeks after IBS-D induction (N = 10).

Experimental data regarding groups 2, 3, were not shown because they did not result in either toxicity or improvement in comparison to sham controls. Furthermore, the IBS-D + BAY 11-7082 10 mg/kg group was only subjected to stool weight, histological evaluation, and MPO activity, because it did not induce any beneficial effect; therefore, we decided to continue analyzing only BAY 11-7082 30 mg/kg.

### 2.5. Restraint Stress Procedure

The restraint stress procedure was performed at the end of experiment as previously described [21]. After fasting for 12 h, each of the IBS-D model and control rats plus treated rats were placed in a restraint cylinder (5 cm × 5 cm × 20 cm), which could limit their body movement but would not cause any breathing problems. The rats remained in the restraint cylinders for 3 h. The feces excreted during restraint stress were collected and weighed. Then, the feces were dried in an oven and weighed again. 

### 2.6. Histological Analysis

Histological analyses were performed as previously described [22]. The colon tissues were fixed with 10% neutral formalin, embedded in paraffin, and sectioned at 7 µm. To detect the epithelial structure and infiltration of inflammatory cells, sections were stained with hematoxylin–eosin (H&E). All stained sections were observed by an inverted microscope with twin CCD cameras (magnification, ×200; Nikon, Tokyo, Japan). The slides were analyzed by a pathologist blinded to the treatment groups. The morphological criteria considered were [23]: 0, normal morphology; 1, minimal scattered mucosal inflammatory cell infiltrates, with or without minimal epithelial hyperplasia; grade 2, mild scattered to diffuse inflammatory cell infiltrates, sometimes extending into the submucosa and associated with erosions; grade 3, mild to moderate inflammatory cell infiltrates that were sometimes transmural, often associated with ulceration, with moderate epithelial hyperplasia; grade 4, marked inflammatory cell infiltrates that were often transmural and associated with ulceration; grade 5, marked transmural inflammation with severe ulceration and necrosis.

### 2.7. Myeloperoxidase (MPO) Activity 

MPO activity, an index of polymorphonuclear cell accumulation, was determined as described by Casili et al. [24]. The rate of change in absorbance was measured spectrophotometrically at 650 nm. MPO activity was measured as the quantity of enzyme degrading 1 mM of peroxide min^−1^ at 37 °C and was expressed in units per gram weight of wet tissue.

### 2.8. Malondialdehyde (MDA) Assay

Malondialdehyde (MDA) level in the colon tissues was determined as an indicator of lipid peroxidation as previously described by Al-Henhena et al. [25]. 

### 2.9. Immunohistochemical Localization of ZO-1, Occludin, IL-1β, IL-18, TNF-α, iNOS, and COX-2

Immunohistochemical localization was made, as previously described by Esposito et al. [22]. Slices were incubated at room temperature overnight with one of the following primary antibodies: anti-zonula occludens-1 (ZO-1) (617300 Invitrogen, Carlsbad, CA, USA, 1:100 in PBS, *v*/*v*), anti-occludin (71-1500 Invitrogen, Carlsbad, CA, USA; 1:100 in PBS, *v*/*v*), anti-tumor necrosis factor-α (TNF-α) (sc-52746 Santa Cruz Biotechnology, Dallas, TX, USA 1:100 in PBS, *v*/*v*), anti-IL-1β (sc-32294 Santa Cruz Biotechnology; Dallas, TX, USA 1:100 in PBS, *v*/*v*), anti-IL-18 (sc-80051 Santa Cruz Biotechnology, Dallas, TX, USA; 1:100 in PBS, *v*/*v*), anti-inducible nitric oxide synthase (iNOS) (610432 BD Transduction, San Jose, CA, USA, 1:100 in PBS, *v*/*v*), and anti-cyclooxygenase-2 (COX-2) (sc-376861 Santa Cruz Biotechnology, Dallas, TX, USA; 1:100 in PBS, *v*/*v*). 

At the end of the incubation with the primary antibody, the sections were washed with PBS and incubated with a secondary antibody (Santa Cruz Biotechnology, Dallas, TX, USA) for 1 h. The reaction was revealed by a chromogenic substrate (brown DAB), and counterstaining with NUCLEAR FAST-RED. A negative control was performed using no primary antibody, particularly, tissue was incubated with the antibody diluent alone, followed by incubation with secondary antibodies and detection reagents. All stained sections were observed and analyzed as previously described. For immunohistochemistry, 20× (50 µm scale bar) and 40× (20 µm scale bar) were shown. Analysis was carried out by assigning quantitative different criteria for staining intensity as described by Ding et al. [26], by using a scale of 0–10 (with 0 indicating a lack of brown immunoreactivity and 10 reflecting intense dark brown staining) and by three different reliable expert observers. The mean was then calculated and results converted into grades: a score of 1–3 was assigned “+”, 4–6 was “++”, more than 7 was “+++”. Scores from all sections of each colon were averaged to give a final score for each rat. All histological studies were performed in a blinded fashion. 

### 2.10. Western Blot Analysis 

Western blot analysis was performed as previously described by Impellizzeri et al. [27]. Protein concentration was estimated by the Bio-Rad protein assay using bovine serum albumin as standard. Samples were then heated at 95 °C for 5 min and equal amounts of protein separated on a 10–15% SDS-PAGE gel and transferred to a PVDF membrane (Immobilon-P). The following primary antibodies were used: anti-nuclear factor of kappa light chain-enhancer in B-cells (NF-κB) (1:500; Santa Cruz Biotechnology, Dallas, TX, USA; sc-8008), anti-inhibitor nuclear factor of kappa light chain-enhancer in B-cells alpha (IκBα) (1:500; Santa Cruz Biotechnology, Dallas, TX, USA; sc-1643), anti-NLRP3 (1:500 Santa Cruz Biotechnology, Dallas, TX, USA, sc-34411), anti-ASC (1:500; Santa Cruz Biotechnology, Dallas, TX, USA sc-22514); anti-caspase1(1:500; Santa Cruz Biotechnology, Dallas, TX, USA; sc-514); anti-iNOS (1:500; 610,432 BD Transduction); anti-COX-2 (1:500; Santa Cruz Biotechnology, Dallas, TX, USA; sc-376861). Antibody dilutions were made in PBS/5% *w*/*v* nonfat dried milk/0.1% Tween-20 (PMT) and membranes incubated overnight at 4 °C. Membranes were then incubated with secondary antibody (1:2000, Jackson ImmunoResearch, West Grove, PA, USA) for 1 h at room temperature. To ascertain that blots were loaded with equal amounts of protein lysate, they were also incubated with β-actin antibody (cytosolic fraction 1:500; Santa Cruz Biotechnology, Dallas, TX, USA) or lamin A/C (nuclear fraction 1:500, Santa Cruz Biotechnology, Dallas, TX, USA). Signals were detected with enhanced chemiluminescence (ECL) detection system reagent according to the manufacturer’s instructions (Thermo Fisher, Waltham, MA, USA). The relative expression of the protein bands was quantified by densitometry with BIORAD ChemiDocTMXRS + software and standardized to β-actin and lamin A/C levels.

### 2.11. ELISA Assay for IL-1β, TNF-α, and IL-18

The levels of IL-1β, TNF-α, and IL-18 were performed by ELISA kit as previously described [24] (Rat IL-1β ELISA kit cat. No. MBS825017 MyBiosource; San Diego, CA, USA); Rat TNFα ELISA kit cat. No. MyBiosource; San Diego, CA, USA; Rat IL-18 ELISA kit cat. No. MBS355269 MyBiosource, San Diego, CA, USA). In details, samples were thawed on ice and homogenized in 300 μL lysis buffer (750 μL, Pierce #87787, Thermo Fisher Scientific, Waltham, MA, USA) supplemented with a protease inhibitor cocktail (Sigma-Aldrich, Rehovot, Israel). Thereafter, the samples were homogenized and centrifuged at 14,000× *g* for 10 min at 4 °C; supernatants were collected, aliquoted, and stored at −20 °C. IL-1β, TNF-α, and IL-18 were measured by ELISA kits according to the manufacturer’s instructions.

### 2.12. Statistical Analysis

The values of each result were displayed as the mean ± standard error of the mean (SEM) of N observations, where N represents the number of animals used. The whole experiment was representative of at least three experiments performed on sections of animals belonging to the various experimental groups. These experiments were conducted on different days. The data were collected and subsequently analyzed by one-way ANOVA, followed by a post-hoc Bonferroni test for multiple comparisons. A *p*-value of less than 0.05 was considered significant.

## 3. Results

### 3.1. Effect of BAY 11-7082 on Restraint Stress

**Figure 1:**
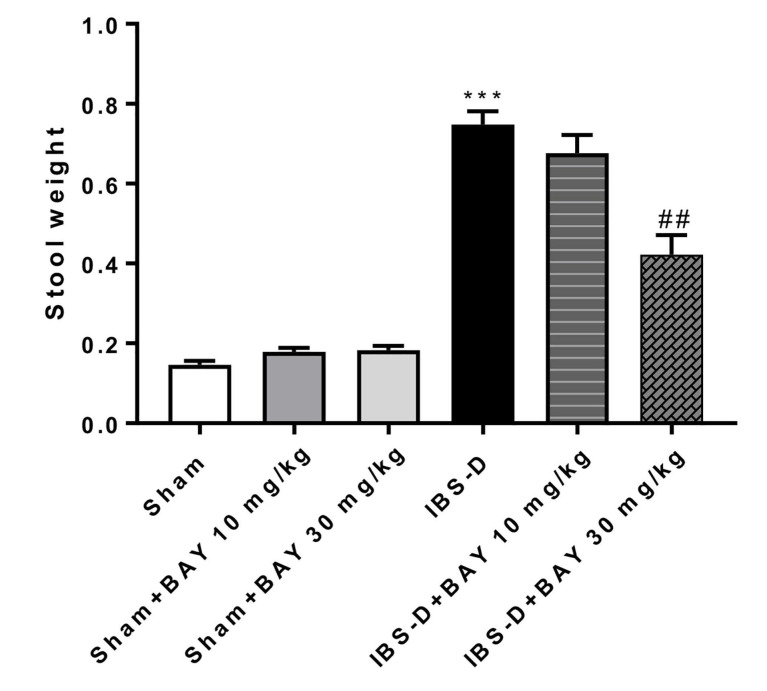
Effect of BAY 11-7082 treatment on restraint stress. The weight of stools was increased by water content in the Diarrhea-predominant irritable bowel syndrome (IBS-D) animals. However, the treatment with BAY 11-7082 30 mg/kg was able to reduce the weight of stools translated in a reduction of stress more than BAY 11-7082 10 mg/kg as shown in Figure 1. Data are representative of at least three independent experiments. *** *p* < 0.001 vs. Sham. ## *p* < 0.01 vs. IBS-D.

The restraint stress procedure was performed at the end of experiment. The results obtained showed that in the IBS-D group, the weight of stools was increased due to water content, while the treatment with BAY 11-7082 30 mg/kg was able to reduce the weight of stools translated in a reduction of stress more than BAY 11-7082 at the dose of 10 mg/kg (Figure 1).

### 3.2. Effect of BAY 11-7082 Treatment on TJs Expression

**Figure 2:**
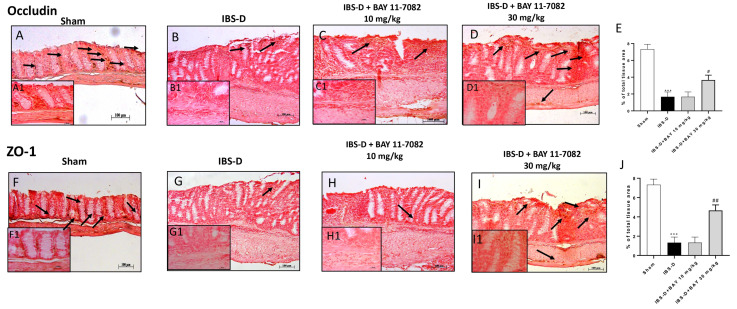
Effect of BAY 11-7082 treatment on TJ expression. Basal expressions of occludin and ZO-1 have been found in the colon tissue of the sham group ((**A**,**F**) respectively) compared to the IBS-D group where a reduction of their expression was evident ((**B**,**G**) respectively). The treatment with BAY 30 mg/kg restored the expression of occludin and ZO-1 to almost basal levels as shown in (**D**,**I**) compared to BAY 11-7082 10 mg/kg (**C**,**H**)). Black arrows display the positive staining. Data are representative of at least three independent experiments. (**E**) *** *p* < 0.001 vs. Sham. # *p* < 0.05 vs. IBS-D. (**J**) *** *p* < 0.001 vs. Sham. ## *p* < 0.01 vs. IBS-D.

Tight junctions (TJs) are multiprotein junctional complexes whose main components are occludin and ZO-1. Occludin and ZO-1 regulate cellular permeability and barrier intestinal function [28]. Disruption of the intestinal TJs represents a critical pathophysiologic factor in patients with bowel disease [29]. Therefore, in this study, we investigated occludin and ZO-1 expression by immunohistochemical staining. Our results showed a basal expression of occludin and ZO-1 in the colon tissue of the sham group (Figure 2A,F); while the IBS-D group was characterized by a reduction of TJs expression (Figure 2B,G). The treatment with BAY 11-7082 at the dose of 30 mg/kg was able to significantly increase occludin and ZO-1 expressions (Figure 2D, see densitometric analysis Figure 2E; Figure 2I, see densitometric analysis Figure 2J), restoring intestinal permeability much more than BAY 11-7082 10 mg/kg treatment that did not show any significance (Figure 2C,H). 

### 3.3. Effect of BAY 11-7082 Treatment on Histological Damage 

**Figure 3:**
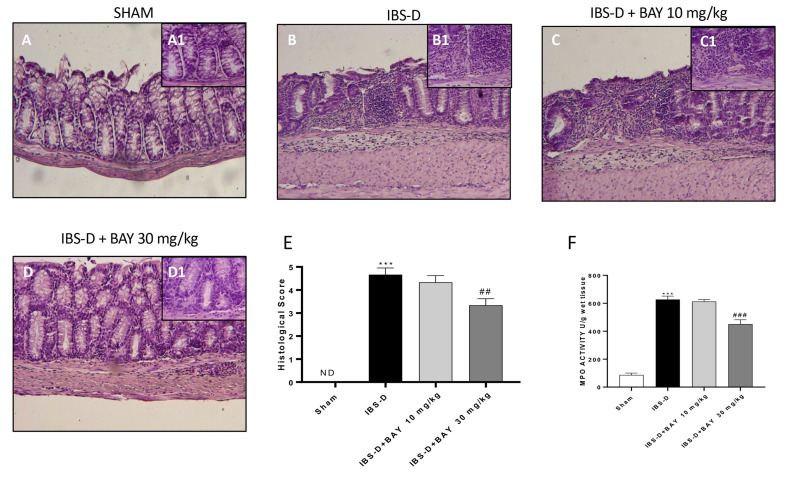
Effect of BAY 11-7082 treatment on histological damage. Extensive damage to the colon tissue was observed in the IBS-D group (**B**) compared to the sham group (**A**). BAY treatment at the dose of 30 mg/kg ameliorated architecture tissue (**D**) more than BAY treatment 10 mg/kg as shown in (**C**). Moreover, as shown in (**F**), the treatment with BAY treatment 30 mg/kg significantly reduced MPO activity more than BAY treatment 10 mg/kg. Data are representative of at least three independent experiments. (**E**) *** *p* < 0.001 vs. Sham. ## *p* < 0.01 vs. IBS-D. (**F**) *** *p* < 0.001 vs. Sham. ### *p* < 0.001 vs. IBS-D.

The histological damage induced by the IBS-D model was evaluated by hematoxylin and eosin staining. A significant tissue damage was observed in the colon tissues from rats subjected to the IBS-D group (Figure 3B), characterized by the loss of tissue structure, edema, and neutrophils infiltration, compared with the sham group where tissue architecture was intact (Figure 3A). The treatment with BAY 11-7082 at the dose of 30 mg/kg significantly ameliorated tissue architecture (Figure 3D) more than BAY 11-7082 treatment at the dose of 10 mg/kg (Figure 3C; see histological score Figure 3E). 

To validate the histological results, we investigated the effect of BAY 11-7082 on the neutrophils infiltration by measuring MPO activity. MPO activity was significantly increased in the IBS-D group compared to the sham group (Figure 3F). However, the treatment with BAY 11-7082 at the dose of 30 mg/kg significantly reduced MPO levels more than BAY 11-7082 10 mg/kg as shown in Figure 3F. 

### 3.4. Effect of BAY 11-7082 Treatment on NLRP3 Inflammasome Pathway

**Figure 4:**
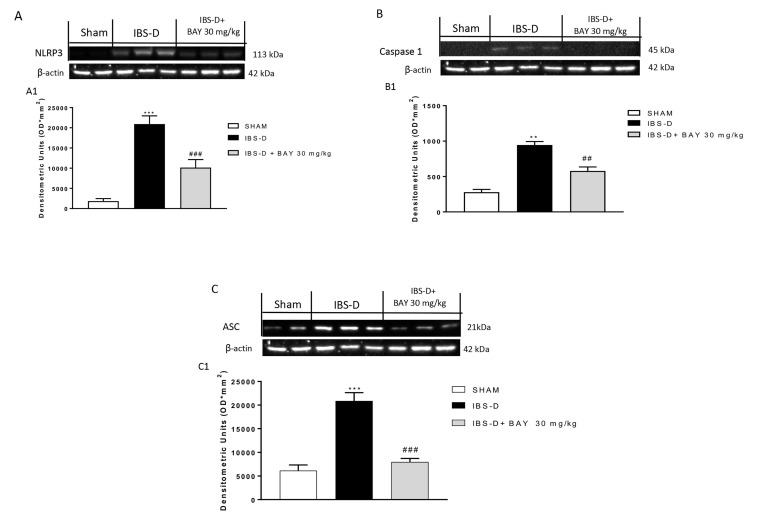
Effect of BAY 11-7082 treatment on the NLRP3 inflammasome pathway. Western blot analysis revealed an increase of NLRP3, ASC, and caspase-1 expression in the IBS-D group compared to the sham group, however the treatment with BAY at the dose of 30 mg/kg significantly reduced their expression ((**A**) and densitometric analysis (**A1**); (**B**) and densitometric analysis (**B1**); (**C**) and densitometric analysis (**C1**), respectively). Data are representative of at least three independent experiments. (**A**) *** *p* < 0.001 vs. Sham. ### *p* < 0.001 vs. IBS-D. (**B**) ** *p* < 0.01 vs. Sham. ## *p* < 0.01 vs. IBS-D. (**C**) *** *p* < 0.001 vs. Sham. ### *p* < 0.001 vs. IBS-D.

The excessive activation of the immune intestinal reaction has been considered one of the main causes of IBS-D development [30,31]. Inflammasome is a cytoplasmic multi-protein complex that regulates innate immunity response through caspase-1 and ASC activation [32]. In this context, NLRP3 appears to play a key role in IBS-D pathophysiology [9]. Therefore, in this study, we investigated the role of NLRP3, caspase-1, and ASC by Western blot analysis. Our results showed that the IBS-D group was characterized by an increase of NLRP3, caspase-1, and ASC expression compared to the sham group; however, the treatment with BAY 11-7082 at the dose of 30 mg/kg significantly reduced their expressions (Figure 4A and densitometric analysis Figure 4A1; Figure 4B and densitometric analysis Figure 4B1; Figure 4C and densitometric analysis Figure 4C1). 

**Figure 5:**
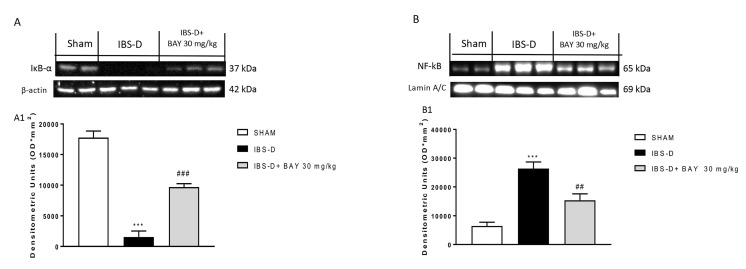
Effect of BAY 11-7082 treatment on the NF-κB/Iκb-α pathway. The blot showed a substantial increase in NF-κB expression (**B**) and a reduction of IκB-α expression (**A**) in the IBS-D group compared to the sham group. The treatment with BAY at the dose of 30 mg/kg significantly reduced NF-κB expression and restored IκB-α expression. Data are representative of at least three independent experiments. (**A**) *** *p* < 0.001 vs. Sham. ### *p* < 0.001 vs. IBS-D. (**B**) *** *p* < 0.001 vs. Sham. ## *p* < 0.01 vs. IBS-D.

Moreover, it has been shown that the NF-κB/Iκb-α pathway is involved in the NLRP3 inflammasome activation promoting the inflammatory response [33,34,35]. Therefore, in this study, we investigated also the role of the NF-κB/Iκb-α pathway by Western blot analysis. The obtained results demonstrated that the treatment with BAY 11-7082 at the dose of 30 mg/kg was able to reduce NF-κB expression (Figure 5B and densitometric analysis Figure 5B1) and restore Iκb-α expression (Figure 5A and densitometric analysis Figure 5A1) compared to the IBS-D group.

### 3.5. Effect of BAY 11-7082 Treatment on Pro-Inflammatory Cytokines, Nitrosative Stress, and Lipid Peroxidation

**Figure 6:**
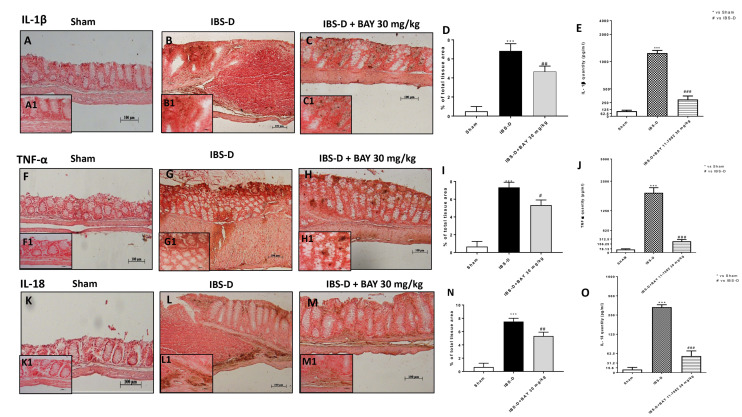
Effect of BAY 11-7082 treatment on IL-1β, TNF-α, and IL-18 expression. By immunohistochemical analysis, an increase of IL-1β, TNF-α, and IL-18 expression was found in the IBS-D group compared to the sham group ((**A**,**B**); (**F**,**G**); (**K**,**L**) respectively); while the treatment BAY 30 mg/kg was able to significantly reduce their expressions ((**C**), see histological score (**D**); (**H**), see histological score (**I**); (**M**), see histological score (**N**)). ELISA kit confirmed the results (**F**,**J**,**O**). Data are representative of at least three independent experiments. (**D**) *** *p* < 0.001 vs. Sham. ## *p* < 0.01 vs. IBS-D. (**I**) *** *p* < 0.001 vs. Sham. # *p* < 0.05 vs. IBS-D. (**N**) *** *p* < 0.001 vs. Sham, ## *p* < 0.01 vs. IBS-D. (**E**) *** *p* < 0.001 vs Sham. ### *p* < 0.001 vs IBS-D. (**J**) *** *p* < 0.001 vs Sham. ### *p* < 0.001 vs IBS-D. (**O**) *** *p* < 0.001 vs Sham. ### *p* < 0.001 vs IBS-D.

Inflammation appears to play a key role in IBS-D development [36]. Therefore, in this study, we investigated, by immunohistochemical staining, the role of pro-inflammatory cytokines as IL-1β, TNF-α, and IL-18 which contribute to increasing the inflammatory process [37]. IL-1β, TNF-α, and IL-18 levels were significantly increased in the IBS-D group compared to the sham group (Figure 6A,B; Figure 6E,F; Figure 6J,K). The treatment with BAY 11-7082 30 mg/kg significantly reduced IL-1β, TNF-α, and IL-18 expressions (Figure 6C, see densitometric analysis Figure 6D; Figure 6H, see densitometric analysis Figure 6I; Figure 6M, see densitometric analysis Figure 6N). The results obtained on IL-1β, TNF-α, and IL-18 were confirmed by ELISA assay as shown in Figure 6E,J,O. 

**Figure 7:**
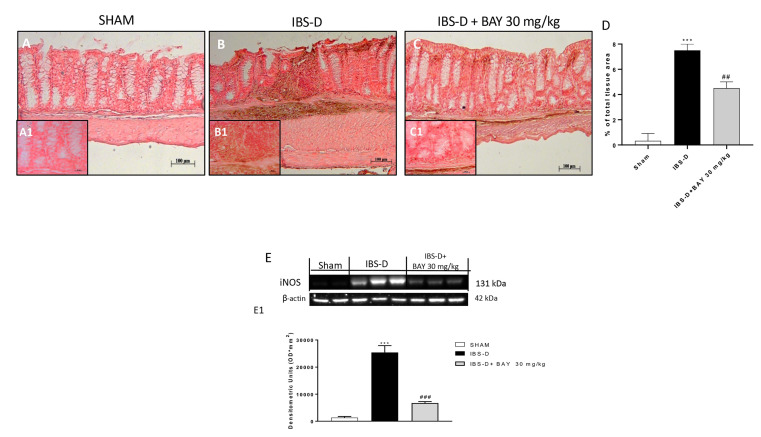
Effect of BAY 11-7082 treatment on iNOS expression. Immunohistochemical analysis revealed an increase of the iNOS level in the IBS-D group (**B**) compared to the sham group (**A**). Moreover, BAY treatment 30 mg/kg significantly decreased iNOS levels (**C**). Data were confirmed by Western blot analysis, showing a decrease of iNOS levels in the IBS-D group following BAY treatment 30 mg/kg. Data are representative of at least three independent experiments. (**D**) *** *p* < 0.001 vs. Sham. ## *p* < 0.01 vs. IBS-D. (**E**) *** *p* < 0.001 vs. Sham. ### *p* < 0.001 vs. IBS-D.

**Figure 8:**
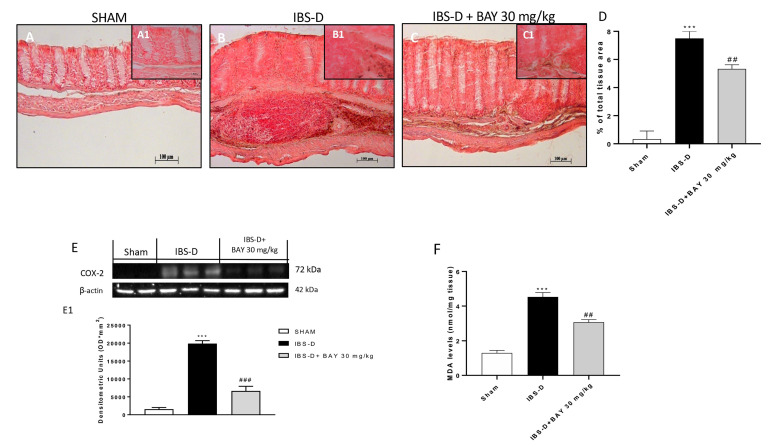
Effect of BAY 11-7082 treatment on lipid peroxidation and COX-2 expression. Immunohistochemical analysis revealed an increase of the COX-2 level in IBS-D groups (**B**) compared to the sham group (**A**). Moreover, BAY treatment 30 mg/kg significantly decreased COX-2 levels (**C**). Data were confirmed by Western blot analysis, showing a decrease of COX-2 levels in the IBS-D group following BAY treatment 30 mg/kg. In addition, BAY treatment 30 mg/kg reduced MDA levels compared to the IBS-D group as shown in (**F**). Data are representative of at least three independent experiments. (**D**) *** *p* < 0.001 vs. Sham. ## *p* < 0.01 vs. IBS-D. (**E**) *** *p* < 0.001 vs. Sham. ### *p* < 0.001 vs. IBS-D. (**F**) *** *p* < 0.001 vs. Sham. ## *p* < 0.01 vs. IBS-D.

Moreover, we investigated nitrosative stress by immunohistochemistry and Western blot of iNOS, one of three key enzymes generating nitric oxide (NO) [38]. In the IBS-D group (Figure 7B and Figure 8B), a positive staining for iNOS compared to the sham group was evident (Figure 7A and Figure 8A), while the treatment with BAY 11-7082 at the dose of 30 mg/kg significantly reduced its expression (Figure 7C, see densitometric analysis; Figure 8C, see densitometric analysis 8D). The result was confirmed by Western blot analysis (Figure 7E and densitometric analysis Figure 7E1).

The lipid peroxidation as well as inflammation is a molecular event involved in intestinal disorders as IBS [39,40,41]. Therefore, considering these evidences, we analyzed MDA levels and COX-2 expression [42]. We performed immunohistochemical and Western blot analysis of COX-2, showing that BAY 11-7082 treatment at the dose of 30 mg/kg was able to reduce its expressions (Figure 8E and densitometric analysis Figure 8E1). Moreover, the IBS-D group was characterized by a significant increase of MDA levels, while the treatment with BAY 11-7082 30 mg/kg significantly reduced MDA levels (Figure 8F).

## 4. Discussion

IBS, as a common functional bowel disorder, is expressed as abdominal pain and/or discomfort and is related with the number and characteristics of bowel movements, but without a clear pathogenesis. Previous studies have proven that inflammation and the immune system are important in the development of IBS-D [43]; it is believed that excessive inflammatory and immune responses in the intestine are due to an imbalance of the intestinal epithelial barrier causing deterioration of the colonic mucus layer which is recurrent in patients with intestinal disorders [44]. Given the multifactorial nature of IBS, currently there is no single management strategy for the different subtypes of IBS, therefore the identification of new molecular targets and alternative treatments is an important goal for research [5]. Scientific evidence shows that NLRP3 plays a regulatory role in the NF-kB activation, contributing to the onset and development of inflammatory disease [45,46], suggesting potential targets for the development of new treatments for patients with IBS. Moreover, the role for NLR inflammasome modulation and caspase-1 activation in mucosal inflammation has been shown to be implicated in IBD and IBS pathophysiology in human [32,47]; a study that compared IBD and IBS-D patients with healthy controls, reported elevated intestinal epithelial cell counts with activated caspase-1 staining [48]. 

Therefore, on these scientific evidences, in this study, we investigated the anti-inflammatory effect of BAY 11-7082, classified as a strong inhibitor of NLRP3, in a vivo model of IBS-D. 

Many research studies revealed that stress aggravates bowel disorders [49,50], in fact, psychological or physical stress is common in patients with chronic gut disorders as IBS-D [51]. High stress levels can lead to a weakening of the immune system, a greater possibility to counteract infections, and a hypersensitivity of the colon promoting the release of pro-inflammatory cytokines [50]. Furthermore, stress can cause a rapid intestinal transit that limits gastrointestinal water absorption, leading to the loss of liquid stools [50]. Stool consistency is considered an important factor to reveal the health of the intestine and the presence of bowel diseases [50]. In this context, our data demonstrated that the treatment with BAY 11-7082 30 mg/kg was able to reduce the water content of stools in the IBS-D group more than the dose at 10 mg/kg, showing the ability to reduce the stress condition.

The intestinal epithelium is an essential permeable barrier essential for nutrient absorption and protection against pathogens [52]. Mucosal barrier is constituted by intestinal epithelial cells and junctions which play an important role in intestinal homeostasis [53]. Several studies showed that alterations of mucosal barrier function are involved in a great variety of intestinal disorders including IBS-D [29,54]. Intestinal epithelial cells are held together by TJs [53], multiprotein complexes which regulate the intestinal permeability by allowing the passage of water, ions, and solutes through pores [27]. Numerous studies confirmed that patients with IBS-D have increased intestinal membrane permeability [53,54], resulting in the visceral hypersensitivity and reduction of TJ-associated proteins ZO-1 and occludin expression [54]. Therefore, TJ disruption likely represents an important aspect of IBS and may be central to the clinical manifestation of diarrhea [6]. Our results confirmed a TJ disruption in the IBS-D group in comparison to the sham group; however, the treatment with BAY 11-7082 30 mg/kg clearly restored ZO-1 and occludin expression to almost basal levels, denoting a restore of intestinal permeability.

IBS-D is characterized by intestinal inflammation condition, associated with polymorph nuclearneutrophilic infiltration (PMN), edema, vasodilation, damage to mucosal glands, and impaired intestinal mucosal integrity [52]. In this context, our study clearly showed significant intestinal morphological changes following IBS-D model induction, accompanied by an increase of neutrophils infiltration. However, the treatment with BAY 11-7082 30 mg/kg restored tissue architecture, reducing neutrophil infiltration also, demonstrating the ability to promote histological recovery following IBS-D. 

IBS patients commonly have NLRP3 expression disorder, rising IL-1β and caspase-1 expressions, and intestinal flora disturbance that further aggravate intestinal inflammation, indicating that NLRP3 dominantly participates in regulation of intestinal flora and maintenance of intestinal homeostasis [55]. In the present study, we demonstrated an increase of NLRP3, ASC, and caspase1 expression in the IBS-D group, however the treatment with BAY 11-7082 30 mg/kg was able to significantly reduce their expression, decreasing NLRP3 inflammasome activity. 

The NLR family member NLRP3 has the ability to facilitate the formation of inflammasome and the activation of mitogen-activated protein kinase (MAPK) and NF-κB signaling cascades, and then initiate and support strong immune responses [56].

The activation of the NF-κB/Iκb-α pathway was discovered to be induced in mucosal macrophages and colonic epithelial cells of IBS patients [57] The abnormal activation of the NF-κB signaling pathway in the intestinal mucosal layer promotes the expression of inflammatory factors, such as NLRP3, which in turn promotes IL-1β and TNF-α production [58]. Induction of the inflammatory cascade and massive neutrophil aggregation can cause damage to intestinal epithelial cell and crypt abscesses formation, leading to an increase in mucosal immune cells and chemokines, followed by an increase in intestinal permeability [59]. In this context, our data confirmed the involvement of the NF-κB/Iκb-α pathway in IBS-D pathogenesis and showed a significant decrease of Iκb-α and an increase of NF-κB expression in the IBS-D group compared to the sham group. However, the treatment with BAY 11-7082 30 mg/kg significantly reestablished NF-κB/Iκb-α expression. 

Consequently, NLRP3 and the NF-κB/Iκb-α pathway, once activated in response to inflammatory stimuli, promote the release of pro-inflammatory cytokines as IL-1β, TNF-α, and IL-18 [56]. In particular, by activating caspase-1, NLRP3 cleaves inflammatory cytokines such as IL-1β and IL-18 and induces immune response by maturing them and releasing them outside cells [60,61]. In our study we showed a marked positive staining for IL-1β, TNF-α, in the IBS-D group, while the treatment with BAY 11-7082 significantly reduced their expression. Moreover, the expression level of IL-18, a downstream cytokine of the NLRP3 inflammasome signaling pathway, was significantly reduced by BAY 11-7082 (30 mg/kg) treatment. 

A strict correlation is observed between NO production and gastrointestinal diseases and it is suggested that elevation of NO plays an important role in the pathogenesis of these pathologies. It has been shown that NO has multiple, potent effects on GI mucosa and may play a role in visceral hypersensitivity and dysmotility in IBS [62]. The obtained results showed an increase of iNOS expression in the IBS-D group compared to the sham group; while the treatment with BAY 11-7082 30 mg/kg was able to significantly reduce their expression, suggesting a reduction of nitrosative stress. 

In recent years, considerable attention has been given to the role of lipid peroxidation in the pathogenesis of IBS [63]. Lipid peroxidation is the result of numerous pathophysiological processes in which oxygen radicals, and in particular MDA, a breakdown product of lipid peroxides, are involved [63]. Because of their ability to generate radicals, and COX-2 [64], due to the accumulation of lipid peroxidation products, they are able to initiate degenerative processes and promote inflammatory bowel disorders by altering intestinal cell membrane integrity [65]. The significant production of MDA in IBS patients suggested that lipid peroxidation, together with oxidative stress, are distinctive markers of this disease [63]. In this context, our data demonstrated an increase of MDA and COX-2 expression in the IBS-D group compared to the sham group; however, the treatment with BAY 11-7082 30 mg/kg was able to significantly reduce lipid peroxidation and COX-2 expression. 

## 5. Conclusions

In conclusion, the obtained data offer new insight into the role of the NLRP3 inflammasome in the onset of IBS-D. As a multifactorial disease, this study has some limitations related to the animal model used which might differ in another model. Clinical models are essential for a fuller understanding of IBS-D onset and progression as well as the activation of inflammatory signaling pathways; probably the development of neuroimaging technology will provide many opportunities for bidirectional translation between IBS patients and IBS animal models.

However, this study clearly demonstrated that the use of a specific NLRP3 inhibitor as BAY 11-7082 (30 mg/kg) could represent a potential therapeutic treatment to counteract and reduce symptoms related to IBS-D, improving the quality of life of patients.
